# Gut Microbiota Are a Novel Source of Biomarkers for Immunotherapy in Non-Small-Cell Lung Cancer (NSCLC)

**DOI:** 10.3390/cancers16101806

**Published:** 2024-05-09

**Authors:** Teresa Del Giudice, Nicoletta Staropoli, Pierfrancesco Tassone, Pierosandro Tagliaferri, Vito Barbieri

**Affiliations:** 1 Department of Hematology-Oncology, Azienda Ospedaliera Renato Dulbecco, 88100 Catanzaro, Italy; vitobarbieri@yahoo.it; 2 Department of Experimental and Clinical Medicine, Magna Graecia University, 88100 Catanzaro, Italy; nicolettastaropoli@gmail.com (N.S.); tassone@unicz.it (P.T.); tagliaferri@unicz.it (P.T.)

**Keywords:** NSCLC, lung cancer, gut microbiota, immunotherapy, immune checkpoint inhibitors

## Abstract

**Simple Summary:**

Lung cancer is the most frequent cause of cancer-related death. Unfortunately, only 30% of patients treated with immunotherapy gain any benefit; it is, therefore, important to increase the number of patients who can benefit from immunotherapy. Biomarkers can help clinicians to reach this target and the gut microbiota is a potentially excellent source of predictive factors. All conditions that modify the gut microbiota may influence cancer onset and progression, its prognosis, and response to immunotherapy, with a relevant impact in clinical practice.

**Abstract:**

Despite the recent availability of immune checkpoint inhibitors, not all patients affected by Non-Small-Cell Lung Cancer (NSCLC) benefit from immunotherapy. The reason for this variability relies on a variety of factors which may allow for the identification of novel biomarkers. Presently, a variety of biomarkers are under investigation, including the PD1/PDL1 axis, the tumor mutational burden, and the microbiota. The latter is made by all the bacteria and other microorganisms hosted in our body. The gut microbiota is the most represented and has been involved in different physiological and pathological events, including cancer. In this light, it appears that all conditions modifying the gut microbiota can influence cancer, its treatment, and its treatment-related toxicities. The aim of this review is to analyze all the conditions influencing the gut microbiota and, therefore, affecting the response to immunotherapy, iRAEs, and their management in NSCLC patients. The investigation of the landscape of these biological events can allow for novel insights into the optimal management of NSCLC immunotherapy.

## 1. Introduction

Lung cancer (LC) is one of the most common causes of cancer-related deaths worldwide. In the past years, new therapeutic approaches have been discovered and, presently, from 15 to 30% of Non-Small-Cell Lung Cancer (NSCLC) patients survive. These patients, in the absence of driver mutations, are treated with immune checkpoint inhibitors (ICIs), alone or in combination, or with chemotherapy. Among ICIs, monoclonal antibodies (mabs) against CTLA4 (cytotoxic T lymphocyte-associated protein 4) and against PD1 (programmed death type 1) or its ligand PDL1 (programmed death ligand type 1) can be used. Ipilimumab, Nivolumab, Pembrolizumab, Cemiplimab, Durvalumab, and Atezolizumab are at present the ICIs used in clinical practice [1]. Not all NSCLC patients benefit from immunotherapy at the beginning of the treatment, while others progress after the initial response to the treatment. At present, a suitable predictive marker for immunotherapy is not available. PDL1, as part of the PD1/PDL1 axis, seems to have a role in predicting the response to immunotherapy, but its expression is inducible and editable by different factors, and, for this reason, its role is challenging, even if it is still the unique predictive biomarker for patient selection by regulatory agencies [2].

Biomarker identification still represents an open challenge for the identification of immunotherapy predictors of response. Examples of different biomarkers include: the neutrophil-to-lymphocyte ratio, tumor-infiltrating lymphocytes, tumor mutation burden, and the gut microbiota [1,2,3]. This latter is composed by all commensal microorganisms present in our gastrointestinal tract, including bacteria, fungi, viruses, and protozoans, while the term microbiome refers to the total genetic material possessed by the microbiota. Alterations in the composition of this microbiota correlate with some diseases, like inflammatory intestinal or metabolic diseases. In the last decades, the gut microbiota has emerged as a crucial player in immunosurveillance and in both cancer onset and progression. In particular, it is possible to hypothesize a gut–lung axis; this theory could explain the correlation between the gut microbiota and an active immune response in LC. The advent of Next-Generation Sequencing (NGS) has allowed for the extensive investigation of the gut microbiome and has correlated it with cancer onset and response to immunotherapy [4].

The correlation between the gut microbiota and immune response in LC could be a potential biomarker in immunity against cancer and particularly in NSCLC patients. It is important to understand how basal microbiota diversity among different patients could affect prognosis and how the gut microbiota could be modified, and how this might change the immune response, and therefore impact the survival of NSCLC patients [5,6]. If this correlation is true, changes to the gut microbiota can potentially improve immunotherapy response, reduce immunotherapy-related adverse events (IRAEs), and prolong survival with immunotherapy treatment [6].

At this time, is important to discover the factors which might influence the gut microbiota, and particularly antibiotics and/or other agents used for different diseases (proton pump inhibitor, antidiabetics as insulin) in cancer patients and specifically in NSCLC patients [7].

**Figure 1:**
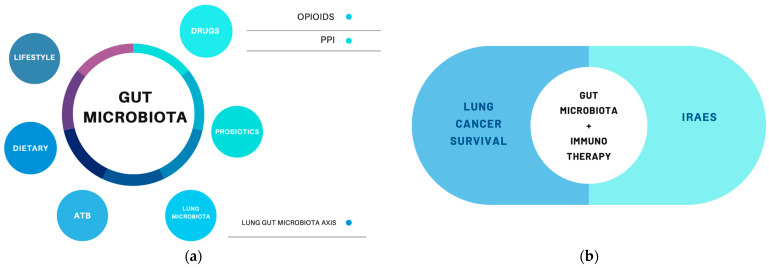
(**a**) Factors involved in microbiota modulation [1,2,3]. (**b**) The gut microbiota’s relevance in LC [7,8,9].

The gut microbiota will be the candidate, given its role in cancer development and immunity, as a new cancer modulator; the objective of this review is to explain, from the current literature, the role of the gut microbiota in LC and how its modulation can improve cancer immunotherapy and IRAE management [6] (Figure 1).

The gut microbiota can be modified by the following different factors: lifestyle, dietary habits, some drugs such as opioids, antibiotics (ATB), PPis, and interactions with other human cells. All this conditions could also be involved, through the modulation of the microbiota, with lung cancer prognosis, survival, immunotherapeutic efficacy, and IRAE development.

## 2. Gut Microbiota and NSCLC

### 2.1. Gut–Lung–Microbiota Axis

The lung microbiota has not been as investigated as the gut microbiota, but its role in different respiratory diseases appears clear [10]. Compared to the gut microbiota, the lung microbiota is smaller, but not less important. The gut microbiota, lung microbiota, and other sites in the human host, in which there are Bacteroides and other elements, are defined as microbial communities [3]. The lung microbiota is composed of *Staphylococcus*, *Streptococcus*, and *Lactobacillus*, followed by *Proteobacteria* and *Actinobacteria*, a microbiota composition similar, in healthy patients, to the gut microbiota. The lung microbiota undergoes changes during inflammation and interacts with metabolites and other pathogens both external and internal to the host [2].

The correlation between the lung and gut microbiotas could depend on the similarity in the mucosa microenvironment, characterized by the same interactions between the microbiota and the immune system in the mucosal immune system (MIS). The MIS is the most important link between these two microbial systems and underlies the participation of the immune system and peptide and protein secretion, such as IgA and metabolite production [3].

Microbiota lung homeostasis is controlled not only within the lung but also by interactions with other organs, and particularly the gut microbiota. The gut microbiota and lung microbiota are linked in many ways: the lymphatic and blood circulation system through the gut microbiota could induce many respiratory diseases, such as asthma, respiratory infection, and others. The gut–lung–microbiota axis is a unique complex that maintains homeostasis; the alteration of this condition can lead to cancer development, tissue damage, and susceptibility to infections [11].

### 2.2. Gut Microbiota Composition, Anti-Tumor Activity, and Antibiotics

The gut microbiota composition seems to be more heterogeneous among different individuals due to different diets, genetic heritages, lifestyles, medical expositions, and other factors. It is clear that its composition correlates with many diseases, including autoimmune disease, inflammatory disease, and cancer [12]. The microbiota composition has an impact on disease pathogenesis, disease prognosis, and the response to therapy. All of these also depend on other factors; for example, it is described that Helicobacter infections are strongly related with gastric adenocarcinoma, but protective for Barret Esophagus development [3,13]. Some data demonstrate that a microbiota enriched with some bacteria, such as Akkermansia muciniphila and Ruminococcacae, correlates with a more favorable outcome in melanoma and NSCLC patients than in head and neck patients, for which the same gut microbiota composition does not modify survival [13]. The presence of Phascolarbacterium is linked to a prolonged Progression-Free Survival (PFS) in NSCLC patients with treatment, while a microbiota enriched with Dialister bacteria in NSCLC patients has a worse prognosis [7]. Patients with a heterogenous gut microbiota composition at baseline have a better prognosis than those patients with a poor heterogeneous composition of microbiota [8]. It appears clear how all conditions modulating the gut microbiota, either with reduced variability or the elimination of good bacteria, can have a negative impact on the prognosis or treatment efficacy for LC patients, suggesting how relevant it is to learn how to modulate them [14].

Recently, some authors have demonstrated, through Mendelian Randomization, a potential correlation among gut microbiota phyla and lung carcinoma subtypes [15]. Three groups of protective microbiotas for the development of NSCLC and nine microbiota groups as risk factors have been identified. However, only one protective intestinal microbiota for the development of small-cell lung cancer (SCLC) and six groups of intestinal microbiotas potentially causing SCLC have been identified. The same authors have just identified some gut microbiota phyla predisposed to lung adenocarcinoma or squamous lung carcinoma. These findings, along with information from the retrospective trial, confirm the correlation between microbiota and lung cancer development, and are also linked to other conditions [15].

LC patients are sometimes treated with antibiotics. There is evidence that the exposure to antibiotics in the first days of life can modify microbiota characteristics, making children susceptible to future inflammatory and autoimmune diseases as compared to non-exposed children. This observation is due to the modification of the gut microbiota composition for months and sometimes for years [16]. It is not surprising, therefore, that antibiotic exposition correlates with cancer onset and progression or immunotherapeutic efficacy [17,18,19].

Potentially, antibiotics can, through the modulation and changing of the composition of the microbiota, reduce and alter the immunotherapeutic activity in LC patients [20]. Numerous data exist in the literature to support how antibiotics, through gut microbiota modulation, could have a negative impact on immune checkpoint activity and chemotherapy activity in cancer and NSCLC patients, and there is evidence that, despite microbiota alterations during antibiotic therapy, no changes in immunotherapeutic efficacy occurred thanks to the ability of the microbiota to return to baseline conditions. The use of antibiotics during immunotherapy in cancer patients could be correlated with primary or secondary immune resistance and, considering that 15–30% of NSCLC patients are treated with antibiotics in clinical practice, the problem is relevant [18].

Studies in mice models demonstrate that the anti-CTLA4 efficacy in cancer patients depends on the gut microbiota composition. A gut microbiota enriched with Bacteroides fragilis or Bacteroides Thetaiotaomicron through polysaccharide products and Th1 response-inducing dendritic cell maturation is related to an improvement in the effectiveness of anti-CTLA4 therapy; moreover, this effectiveness is restored by diet or the oral supplementation of this bacterium. At the same time, Bifidobacterioides improve anti-PD1 efficacy in melanoma-affected mice, while mice with a different gut microbiota, but undergoing fecal Bifidobacterioides-based microbiota transplantation, become anti-PD1 responders thanks to an increased T cell anti-tumor response [6].

Since 2017, several retrospective studies about the antibiotic effect on lung cancer patients treated with immunotherapy have been reported. The data from these trials are not completely consistent and the antibiotic role remains unclear. Particularly, some studies demonstrate a correlation between antibiotic exposure and worse prognosis and reduced immunotherapy efficacy, while other studies do not demonstrate such correlation, which could be associated to several factors, some associated to the host microbiota and host characteristics, and others to a selection study bias (Table 1).

**Figure 2:**
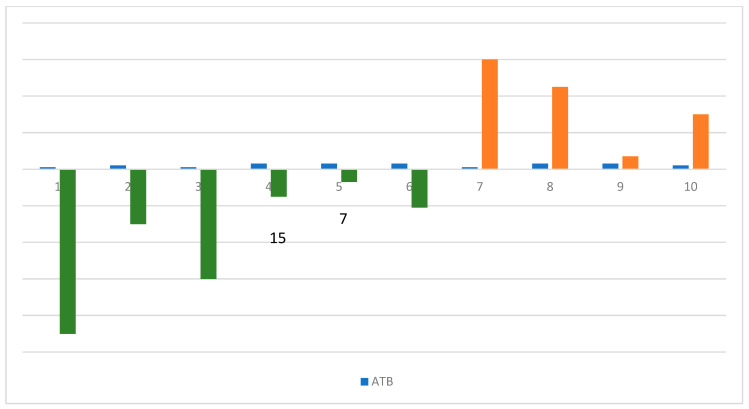
This figure explains the correlation between antibiotics (ATBs), shown with the blue bar, and its exposure time before (green bar) and after (orange bar) ICI (immune checkpoint inhibitor) treatment. The role of Fluoroquinolones (1) in ICI efficacy is evaluated at 90 and 60 days before the start of treatment. Fluoroquinolones, Vancomycin, Piperacillin–Tazobactam, Clindamycin, and other not-specified ATBs (2, 3) are evaluated at an exposure time of 30 days before and after the start of the ICIs. Finally, a group of not-specified ATBs (5, 6, 7, 9, 10) are evaluated in many exposure timings: 21, 15, and 7 days before and 45 and 7 days after.

**Figure 3:**
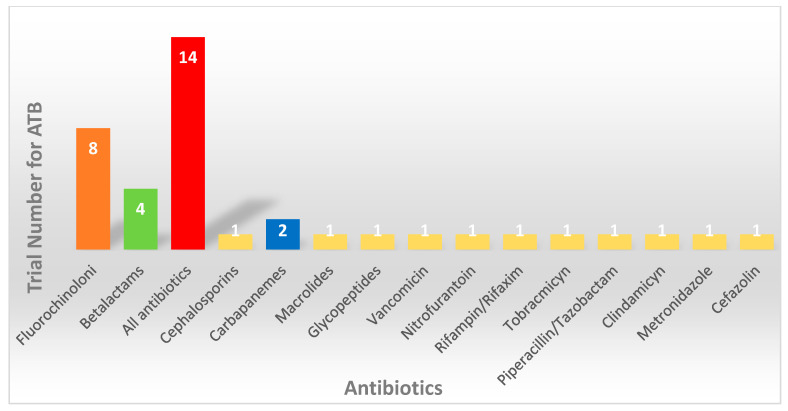
The different antibiotic (ATB) types evaluated in retrospective trials identified in the literature are reported. A total of 14 trials did not specify the ATB used (red bar), while Fluoroquinolones are the most represented (orange bar). The other ATBs are evaluated in poor retrospective trials (green bar, blue bar, and yellow bars). Several ATBs are evaluated, but the resulting data are controversial.

The time to antibiotic exposure and different antibiotic types is reported in Figure 2 in order to describe the detrimental effect on the immunotherapeutic efficacy due to microbiome alteration. Figure 3 reports on the different antibiotics evaluated in retrospective studies with respect to correlations to immunotherapy efficacy.

The role of antibiotics in cancer treatment is controversial: antibiotics are essential for managing infections but their indiscriminate use can disrupt the gut microbiota, potentially impairing the effectiveness of immunotherapies in cancer patients, including those with NSCLC. However, we also acknowledge the conflicting data and the complex factors at play, including the ability of the microbiota to return to baseline conditions after antibiotic treatment and the varied impacts depending on the patient’s unique microbial composition [19,20].

**Table 1:** Retrospective studies from 2017 to 2023, including lung cancer patients treated with immunotherapy and those exposed to antibiotics.

Year	Author	Patients	Treatment	Antibiotic Typologies	Antibiotic Exposistion Timing	Reference
2017	Kaderbhai et al.	74	Anti-PD1, Nivolumab	Fluoroquinolones	3 months before starting ICIs	[21]
2018	Derosa et al.	239	Anti-PD1, Anti-CTLA4 Monotherapy or combination	Fluoroquinolones, Betalactams	30 days before starting immunotherapy	[22]
2018	Hakozaki et al.	90	Anti-PD1, Nivolumab	Not specified	30 days before starting immunotherapy	[18]
2018	Huemer et al.	30	Anti-PD1, Nivolumab, Pembrolizumab	Not specified	30 days before and after starting immunotherapy	[23]
2019	Zhao S et al.	109	Anti-PD1, Anti-PDL1	Not specified	Not specified	[24]
2019	Kim H et al.	131	Anti-PD1, Anti-PDL1, Anti-CTLA4 Monotherapy or combination	Fluoroquinolones, Betalactams, Cephalosporins	60 days before starting immunotherapy	[19]
2019	Galli et al.	157	Anti-PD1, Anti-PDL1, Anti-CTLA4 Monotherapy or combination	Not specified	Before and during immunotherapy	[25]
2020	PH Lu et al.	340	Anti-PD1, Anti-PD1, Anti-CTLA4 Monotherapy or combination	Fluoroquinolones	30 days before starting immunotherapy	[26]
2020	E Pérez-Ruiz et al.	120	Anti-PD1, Anti-CTLA4 Monotherapy or combination	Not specified	Not specified	[27]
2020	Svaton M et al.	224	Anti-PD1, Nivolumab	Not specified	Not specified	[28]
2020	Chalabi M et al.	757	Anti-PDL1, Atezolizumab	Fluoroquinolones, Carbapanems, Macrolides, Glycopeptides	30 days before and after starting immunotherapy	[29]
2020	Tinsley et al.	64	Anti-PD1	Not specified	15 days before and 45 days after starting immunotherapy	[30]
2020	Kulkarni et al.	140	Anti-PD1	Vancomicyn, Nitrofurantoin, Rifampin, Rifaximin, Tobramicyn	30 days before and after starting immunotherapy	[31]
2021	Geum et al.	140	Anti-PD1, Nivolumab	Not specified	Not specified	[32]
2021	Cortellini et al.	302	Chemotherapy, Immunotherapy	Not specified	7 days before and after starting immunotherapy	[33]
2021	Giordan et al.	65	Anti-PD1, Anti-CTLA4, Monotherapy or combination	Not specified	60 days before starting immunotherapy	[34]
2021	Cortellini et al.	950	Anti-PD1, Pembrolizumab	Piperacillin-Tazobactam, Clindamycin, Metronidazole, Meropenem	30 days before starting immunotherapy	[35]
2021	Hamada et al.	69	Anti-PD1	Not specified	21 days before starting immunotherapy	[36]
2022	Hopkins et al.	2723	Anti-PDL1, Atezolizumab	Not specified	30 days before starting immunotherapy	[37]
2022	Barbarosa et al.	140	Anti-PD1, Anti-PD1, Anti-CTLA4, Monotherapy or combination	Fluoroquinolones, Betalactams	2 months before and after starting immunotherapy	[17]
2022	Nyein et al.	256	Anti-PD1, Anti-PDL1, Anti-CTLA4, Monotherapy or combination	Fluoroquinolones, Cefazolin, Azithromicin	60 days before and after starting immunotherapy	[38]
2022	Qiu H et al.	148	Anti-PD1, Anti-PDL1, Chemotherapy	Fluoroquinolones, Betalactams	60 days before and after starting immunotherapy	[39]
2023	Manning-Bennett et al.	2724	Anti-PDL1, Atezolizumab, Alone or in combination with chemotherapy	Not specified	Not specified	[40]
2023	Vihinen et al.	199	Anti-PD1, Anti-PDL1	Not specified	3 months before and 1 months after starting immunotherapy	[41]

A retrospective analysis of 70 NSCLC patients treated with ICIs investigated the gut microbiota diversity in patients with an OS (Overall Survival) >12 months and <12 months. The gut microbiota of long survivors was enriched with *Lachnospiraceae*, a member of *Clostridiale*, with increased circulating CD4 and CD8 T cell and CD8 T cell-infiltrating tumors. The study confirmed that a diversified microbiota correlates with a better prognosis and that the use of antibiotics also reduces the diversity of the gut microbiota [42].

The antibiotic exposure from 60 days before starting immunotherapy and 30 days after the last immunotherapy correlated with a poor prognosis and immunotherapy resistance [14]. Scarce information on the antibiotic type, antibiotic route, and the duration of therapy are available. Greater information would help us to use microbiota modulation to improve immunotherapy in cancer. As just mentioned, the microbiota has the ability to return to homeostasis after antibiotic damage: different time frames of the reconstitution of the baseline status might explain the unclear data on the prognosis and exposure to antibiotics in different patients with lung cancer [11,13,16].

There is evidence about the relevance of the gut microbiota on the efficacy and toxicity of chemotherapy and the intestinal microbiota; the maximum effectiveness of chemotherapy in treated cancer is mediated by a good balance between the intestinal microbiota and the immune system [3]. The relevance of the gut microbiota in chemotherapy management and efficacy represents today an important issue considering NSCLC treatment based not only on ICI monotherapy but also on the chemo–immunotherapy association [43,44].

It is clear that the microbiota have a role in cancer from onset to therapy response, but the knowledge of the conditions implicated in the change in the intestinal microbiota that should be avoided unless necessary, such as the use of antibiotics, remains to be clarified [8,13,20].

### 2.3. Gut Microbiota and Probiotic Use

Since the modulation of the gut microbiota can modify the effectiveness of immunotherapy in cancer patients, finding a way to remodulate the microbiota and restore it so to improve the effectiveness of immunotherapy could be an option for our patients. Oral probiotic supplements have been associated with the improved efficacy of immunotherapy for cancer patients [45,46]. Probiotics are a bacterial strain that do not alter antibiotic resistance; they reach the colon and the entire intestine, where they carry out their metabolism. Probiotics may be safe in animals, resistant to acids, and able to colonize the intestine [3]. Probiotics can modulate the gut microbiota by (a) modifying humoral, cellular, and innate immunity; (b) improving NK (Natural Killer) immune activity; (c) macrophage and neutrophil activation; (d) IgA secretion; (e) inflammatory cytokine inhibition. Moreover, probiotics can modulate chemotherapy toxicity and iRAEs development. To date, the more used probiotics are composed of *Bifidobacterium* spp. and *Lactobacillus* spp.; their role appears marginal in NSCLC patients treated with immunotherapy because they are not specifically chosen for this reason. The discovery of probiotics able to modulate the immune response with immunotherapy and able to prevent and improve iRAEs and chemotherapy toxicities [3], or limit the unfavorable effect of antibiotics [47], could be very interesting.

With the recent advent of Next-Generation Sequencing (NGS), new species of probiotics have been identified and called next-generation probiotics (NGPs), which are presently under evaluation in the context of specific diseases. NGPs are able to modulate the gut microbiota to improve the immunotherapy and control iRAEs. *Eubacterium limosum*, *E. hirae*, *Enterococcus faecium*, *Collinsella aerofaciens*, and *Burkholderia cepacia* appear to have promising efficacy in this setting [3].

A recent metanalysis underlined the role of probiotics and their effect on the survival of NSCLC patients treated with immunotherapy. This study demonstrated a positive correlation between probiotic exposure and OS and PFS. There was no correlation with ORR, but it can be demonstrated by the types of studies and the sample size [46].

Probiotic use can improve immunotherapeutic efficacy through the modulation of inflammation. The data about this correlation are limited by study design, cancer types, sample size, and the duration of oral probiotic implementation [46]. Certainly, the use of probiotics increases the heterogeneity of the intestinal microbiota, which is the basis of a better prognosis, a better response, and fewer iRAEs in cancer patients [9]. It is important to understand whether a single bacterial species can modulate the entire microbiota or if the presence of different bacterial species is necessary at the same time. It is also important to understand the amount of single species that are needed for a beneficial effect to a well-balanced gut microflora [8].

### 2.4. Gut Microbiota and iRAEs

In the era of immunotherapy, in which cancer patients undergo ICI treatment for long periods, toxicity becomes the most relevant issue. Few data have evidenced correlations between the gut microbiota composition and iRAEs: some bacterial strains seem to be protective against iRAEs, while other strains could increase the risk of iRAEs [48].

In a retrospective analysis, a link between the gut microbiota, antibiotic exposure, and iRAEs was investigated: a correlation between antibiotic use and iRAEs was not observed, but a gut microbiota enriched with Akkermansia Mucinipihila correlated with fewer iRAEs [42].

Microbiota diversity correlates with the development of iRAEs during immunotherapy. As demonstrated for the survival and prognosis, it appears that patients with a low diversity of gut microbiota exhibit skin iRAEs more often than patients with a gut microbiota enriched with many bacterial types [1,9].

In the gut microbiota composition of mouse models and cancer patients, the presence of Bacterioides and other microbes implicated in vitamin B production seem to be protective against colitis development during immunotherapy; the detection of some types of Bacterioides in the gut microbiota can help us to predict colitis presentation during immunotherapy, while bacterial supplementation with *Bacteroidales* and *Burkholderiales* can improve colitis, particularly in cancer patients treated with antibiotics during immunotherapy [48].

The gut microbiota composition could be implicated in skin iRAEs during immunotherapy in cancer. This theory is supported by modulatory effects on the skin mediated by the gut microbiota through immunity regulation and metabolite products. There is evidence of the improvement of dermatitis with the use of oral Lactobacillales and Bifidobacteriales. The oral use of Bifidobacteriodes in humans reduces inflammatory markers such as peptide C and TNF-alpha, with an improvement in psoriasis [9]. It is necessary to gain more information and data about the gut microbiota and iRAEs to improve immunotherapy management [9].

### 2.5. Other Conditions Modifying Gut Microbiota

In addition to the role of antibiotics and probiotics in the modulation of the intestinal microbiota for the improvement in the efficacy of ICIs in LC patients, there are other molecules that are potentially implicated and that have been recently studied, with more hypotheses and a few relevant points that need to be confirmed by other studies.

One of the retrospective studies in the literature about gut microbiota modulation mediated by drugs, describing 132 lung patients treated with immunotherapy, presents a shorter PFS and OS when exposed to opioids through the impairment of T cell function, upregulating Treg cells modulating the gut microbiota; the opioid exposition did not correlate with different iRAE incidence. This exploratory data could be important in considering the high percentage of cancer and LC patients exposed to opioid drugs for pain management, and this research area needs to be focused on. It could be very important to acquire data to confirm these results, considering that pain is one of the most important causes of quality-of-life reduction, and that this could be correlated to a worse response to immunotherapy. This could mean, as suggested in a small study, that worse PFS and OS are not related to opioid exposure but to a poor Performance Status (PS) [49].

In 2021, one of the first retrospective papers about the correlation between PPi (Proton Pump Inhibitor) exposition and LC patient survival treated with immunotherapy was presented. Lung cancer patients treated with ICIs and exposed to PPis have a 28% increased risk of death and a shorter survival compared to unexposed patients; this relationship is not observed in subgroup patients exposed to PPis but treated with chemotherapy only. The PPi/survival association is consistent considering the sample size, and the data are confirmed when the exposition window changes. The possible cause of this negative relationship can be linked to PPi-mediated acid reduction, which alters the intestinal microbiota [50]. Recently, another author published a metanalysis regarding PPi exposition and survival in cancer patients treated with immunotherapy that confirmed the negative relationship; also, in this case, the relationship could be modified by the poor quality of life in cancer patients exposed to PPis [51]. In both works presented, there are many limitations; one of the limitations is the presence of retrospective trials, as well as the PPi exposition (dose, time, and PPi use). However, other retrospective studies and metanalyses did not confirm the relationship between PPis and immunotherapy efficacy [50,51].

## 3. Future Directions

Despite the recent huge impact of cancer immunotherapy, only 30% of lung cancer patients gain any benefit. This condition depends on different factors that are intrinsic and extrinsic to LC patients [11]. For these reasons, it is relevant to identify biomarkers to select responder patients, improve the immunotherapeutic efficacy, and manage iRAEs. Among the well-known biomarkers, such as PDL1 and the PD1–PDL1 axis, TMB (tumor mutational burden), and others, the microbiota potentially appears to be the most relevant predictor of immunotherapeutic efficacy. There are many factors that can influence and modulate the microbiota, and all of them could play an important role in cancer development as well as in the efficacy and toxicity of immunotherapy [48,52].

Interestingly, among the numerous factors implicated, several authors have demonstrated that different bacteria defined as “intratumoral microorganisms” appear to be related to the progression of different types of cancer. Bacteria were detected both in immune cells and tumor cells, determining a strong tumor microenvironment involvement. Recent data confirmed a potential prognostic role of “intratumoral microorganisms” and the potential therapeutic implications [53].

The evaluation of the “intratumor microbiome” was conducted with several methodologies based on the deep sequencing of the amplicons of prokaryote and eukaryote rRNA genes or metagenome-based shotgun sequencing (WMS). Moreover, immunohistochemistry (IHC), fluorescent in situ hybridization (FISH), and D-alanine-based methods have allowed authors to evaluate the presence and characteristics of specific tumor bacteria. Finally, the QIIME 2 method was described as a reproducible “intratumoral” microbiome data analysis system [54].

The intestinal microbiota plays a role in anti-tumor immunotherapy and, therefore, all conditions that modify the intestinal microbiota, reducing its diversity, might potentially modify the efficacy and also the occurrence of iRAEs. We have relevant information about the microbiota–immunotherapy relationship, but we have no clear-cut information that presently allows us to improve our approach to cancer therapy management [10,48]. It is not only important to know the composition of the microbiota but also the interplay between all players of the intestinal microbiota and the microenvironment that surrounds it, taking into account that all elements are regulated by different intrinsic and extrinsic factors, including the genetic host features, diet, lifestyle, age, and concomitant medication [10]. This is why the microbiota cannot be considered a weapon to be used, but rather a biological entity whose relevance is still underestimated.

More controversial findings focus attention on the potential role of microbiota transplantation in order to influence the tumor microenvironment and the “intratumoral microbiome”. However, no evidence supports this practice to improve immunotherapeutic efficacy [55,56].

At present, what we know about the microbiota and its relationship with cancer immunotherapy is derived from retrospective analyses or meta-analyses based on retrospective studies. All derived information could be defined as hypothesis-generating and not clear-cut findings, which can be derived only by prospective and randomized trials. For example, we know that the exposure to antibiotic exposition can adversely modulate the gut microbiota, thus reducing the efficacy of immunotherapy [25]. However, we do not know which class of antibiotics are implicated, the timing of the exposure, or the differences in the routes of administration; at the same time, we know that not all immunotherapy-treated patients have a worse prognosis related to exposure, and many sources of biases can be identified. The same difficulty occurs in understanding the gut microbiota–immunotherapeutic efficacy relationship with opioids and PPis, whose role has always been investigated retrospectively.

Therefore, the more relevant question is the following: what is the next step for translating microbiota knowledge into LC management?

Again, a major role is played by the retrospective study design versus prospective trials, which are pivotal trials for the adequate stratification of the administered drugs.

Considering the huge world represented by the microbiota, it is clear that the current methodologies are not completely applicable and new methodologies might be considered in the future. The machine learning (ML) recently used in cancer immunotherapy for predicting the development of iRAEs in cancer patients during immunotherapy [57] could help us to identify the role of the exposure to many drugs through microbiota modulation. Instead of predicting iRAEs, modifying the microbiota conditions depends on various elements which are not simple to manage in clinical practice: for this reason, AI (artificial intelligence) and ML could help us in our objective. There are several reports about using ML and microbiota. From the data on the microbiota produced by omics-based methods (metagenomics, meta transcriptomics, and metabolomics), ML can predict and find new non-theoretically inferred information to help us to increase the efficacy and reduce iRAEs [58,59].

Alongside AI and ML, which could be of help in the future on this topic, it needs to be highlighted that microbiota and microbiomes not are the same in all populations, in all persons, and in all cancers. We know today about LC microbiota and that its correlation with immunotherapy efficacy is different from head and neck microbiota, SCLC microbiota, or bladder microbiota. It is important to make clear that the relevance of microbiota in LC does not necessarily translate in other cancers which grow in a different tumor microenvironment.

## 4. Conclusions

To date, the intestinal microbiota appears to be an important biomarker of immunotherapy in LC. However, taking into account the complexity of the whole scenario, it is necessary to make a great effort to gain more functional information. It is also necessary that the data available are confirmed by more robust trials or in an alternative way through new methodologies, such as AI. While waiting for all of this information, what we can tell from the microbiota, cancer prevention, and immunotherapy is that it is important to limit the use of antibiotics, PPis, and opioids, which can be used if necessary, and that probiotic use could improve some conditions such as colitis management and other iRAEs. More adequate probiotics might be identified with NGS and NPS. A healthy diet and a better lifestyle might be, in any case, the mainstream proposal to our patients.
